# Retraction: Oncogenic extracellular HSP70 disrupts the gap-junctional coupling between capillary cells

**DOI:** 10.18632/oncotarget.28812

**Published:** 2025-12-31

**Authors:** Dominique Thuringer, Kevin Berthenet, Laurent Cronier, Gaetan Jego, Eric Solary, Carmen Garrido

**Affiliations:** ^1^INSERM, U866, Faculty of Medecine, Dijon, France; ^2^CNRS ERL7368, STIM Lab, University of Poitiers, Poitiers, France; ^3^University of Burgundy, Dijon, France; ^4^INSERM, U1009, Institut Gustave Roussy, Villejuif, France; ^5^CGFL, BP77980 21000 Dijon, France

**This article has been retracted:** This decision follows an investigation by Oncotarget journal regarding the concerns raised by the readers on PubPeer. Our image forensics analysis revealed multiple issues of internal and external duplications and overlaps:

- Figure 1A: FRAP images of HMEC cells were reproduced in Figure 2A of a concurrently published paper from the same lab [1] without reference or explanation.
- Figure 2C and 2F: The Western blot for p-Ser-255 of Cx 43 in Figure 2C overlaps with the ZO-1 blot in Figure 2F, representing different experimental conditions.
- Figures 2C, 6A, and 6B: The Western blot for Hsc70 was used three times (after horizontal flip and/or change in aspect ratio) to represent different experiments.
- Figure 3A: The Western blot for EGFR was previously published in Figure 4A of a 2011 paper from the same lab [2] but illustrated a different experiment.
- Figure 3B: The Western blot for EGFR P-Tyr was previously published as a blot for P-NF-kB p65 in Figure 4E of a 2013 paper from the same lab [3], which was recently retracted.
- Figure 7A and 7D: Previously corrected cell images in Figure 7D (overlapped with images in Figure 6F control shRNA) have an overlapping field of view with images in Figure 7A.

Although the authors provided the Editorial Office with corrected Figures 2C, 6A, 6B, and 7D, they failed to provide uncropped original blots and cell images with date stamps.

Taking into consideration the number of necessary corrections and image manipulations, the Editorial decision has been made to retract this paper. The authors have been informed of this decision.

Original article: Oncotarget. 2015; 6:10267–10283. 10267-10283. https://doi.org/10.18632/oncotarget.3522
